# High-density isochronal repolarization mapping and re-entry vulnerability estimation for scar-related ventricular tachycardia ablation: mechanistic basis, clinical application, and challenges

**DOI:** 10.1093/europace/euae271

**Published:** 2024-10-31

**Authors:** Johanna B Tonko, Anthony Chow, Pier D Lambiase

**Affiliations:** Institute for Cardiovascular Science, University College London, 5 University Street, London WC1E 6JF, UK; Barts Heart Centre, St Bartholomew’s Hospital, W Smithfield, London EC1A 7BE, UK; Barts Heart Centre, St Bartholomew’s Hospital, W Smithfield, London EC1A 7BE, UK; Institute for Cardiovascular Science, University College London, 5 University Street, London WC1E 6JF, UK; Barts Heart Centre, St Bartholomew’s Hospital, W Smithfield, London EC1A 7BE, UK

**Keywords:** Re-entry vulnerability, Repolarization mapping, Isochronal crowding, Ventricular tachycardia, Functional substrate mapping, Deceleration zones, Line of block

## Abstract

Alterations in repolarization gradients and increased heterogeneity are key electrophysiological determinants of ventricular arrhythmogenesis across a variety of aetiologies with and without structural heart disease. High-density repolarization mapping to localize these repolarization abnormalities could improve characterization of the individual arrhythmogenic substrate and inform more targeted ablation. Yet, due to challenges posed by intrinsic features of human cardiac repolarization itself as well as technical and practical limitations, they are not routinely assessed, and traditional substrate mapping techniques remain strictly limited to determining conduction abnormalities. Here, we provide an overview of the mechanistic role of repolarization alterations in ventricular re-entry arrhythmias followed by a description of a clinical workflow that enables high-density repolarization mapping during ventricular tachycardia (VT) ablations using existing clinical tools. We describe step-by-step guidance of how-to set-up and generate repolarization maps illustrating the approach in case examples of structural normal and abnormal hearts. Furthermore, we discuss how repolarization mapping could be combined with existing substrate mapping approaches, including isochronal late activation mapping, to delineate sites of increased re-entry vulnerability, that may represent targets for ablation without the requirement for VT induction. Finally, we review challenges and pitfalls and ongoing controversies in relation to repolarization mapping and discuss the need for future technical and analytical improvements in repolarization mapping to integrate into ventricular substrate mapping strategies. Repolarization mapping remains investigational, and future research efforts need to be focused on prospective trials to establish the additional diagnostic value and its role in clinical ablation procedures.

## Graphical Abstract

**Graphical Abstract:**
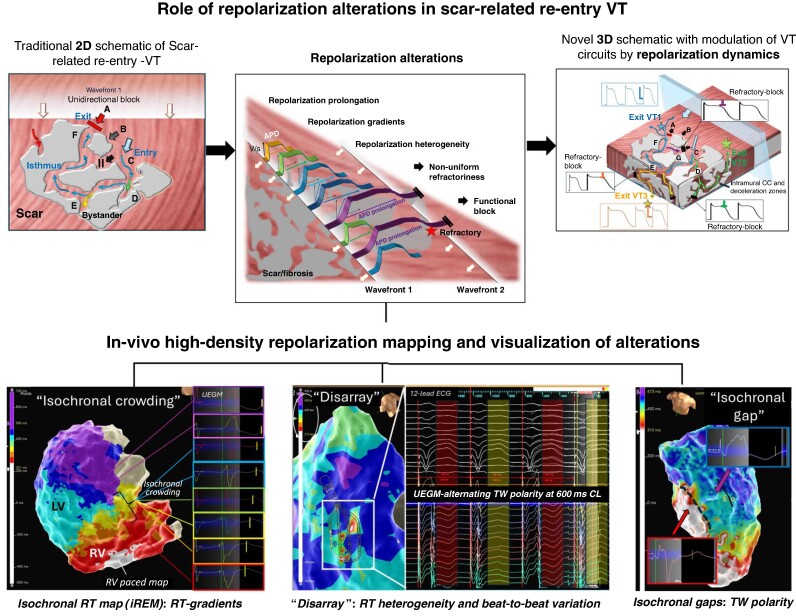
Role of repolarization alterations in scar-related re-entry and assessment by electro-anatomical mapping.

**What’s new?**

- High-density repolarization mapping is feasible using existing mapping technology and can inform about ventricular repolarization gradients and heterogeneities.
- Assessment of repolarization dynamics may provide important complementary information during ventricular substrate mapping to identify targets for ablation if employed in combination with conduction metrics to locate sites of high re-entry vulnerability.
- The restriction to unipolar electrogram and thus lower spatial resolution and susceptibility to noise remain important challenges, as are the difficulties in capturing temporal dynamic and changing beat-to-beat phenomena with contemporary mapping technology.

## Introduction

Ablation is an established treatment modality for patients with ventricular arrhythmias to reduce arrhythmia burden and prevent ICD therapies. Numerous mapping approaches have been proposed over the years to identify ventricular ablation targets. They have evolved from traditional activation and entrainment mapping during ventricular tachycardia (VT) to substrate mapping assessing distinct voltage-, timing-, or morphology-based electrogram features during sinus or paced rhythms to more recently functional mapping strategies with a variety of pacing protocols aiming to unmask decremental conduction and/or evaluating the substrate during multiple wavefronts.^1^ A common feature across all these mapping approaches is their exclusive focus on detecting conduction alterations while disregarding pathological repolarization changes. However, repolarization heterogeneities and abnormally steep gradients play an important pathophysiological role in ventricular arrhythmogenesis in both structural^2,3^ and primary electrical cardiac disorders.^4,5^ Technical and practical aspects of clinically available mapping equipment have so far prevented routine assessment of repolarization dynamics during clinical procedures. Yet, assessment of conduction metrics alone may incompletely characterize arrhythmogenic susceptibility of the substrate since it is likely the dynamic *interplay* between conduction and repolarization dynamics, which results in the initiation and maintenance of re-entry arrhythmias.^3,6^ Integrated assessment would thus be key to identifying sites of increased re-entry vulnerability, and a quantified re-entry vulnerability index (RVI) delineating isthmus exit sites without the requirement of VT induction has been described.^7,8^ Translating these findings of preclinical and clinical retrospective analyses into real-time clinical practice could improve current substrate mapping and also, potentially, procedural outcomes.

## The role of repolarization abnormalities in re-entry arrhythmias

### Physiology

**Figure 1:**
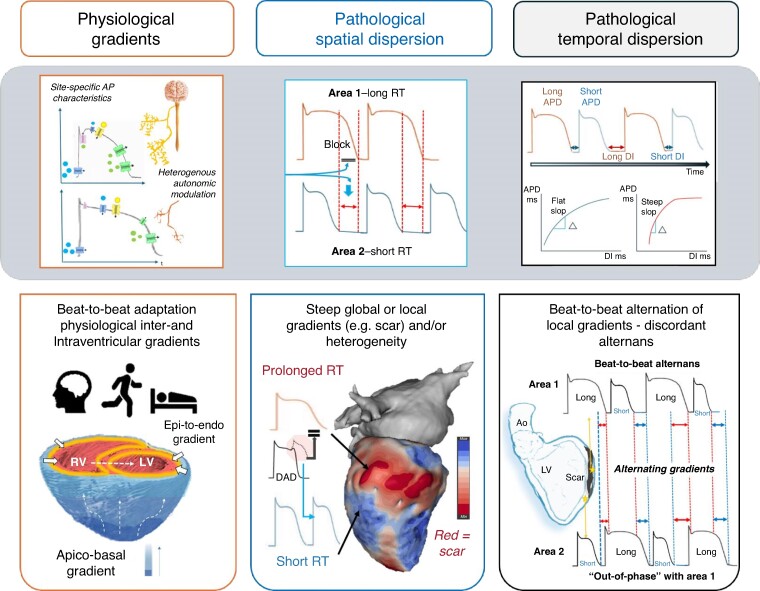
Physiological repolarization gradients in the heart and normal beat-to-beat adaptation based on physiological demands. Under pathological conditions, both spatial dispersion and temporal dispersion of repolarization are accentuated and steep gradients as well as sites of high RT heterogeneity may occur and increased the susceptibility to ventricular arrhythmias. AP, action potentials; APD, action potential duration; DI, diastolic interval.

Repolarization gradients, defined as differences in repolarization time along a specific axis (e.g. transmural, apico-basal), are known to exist in the human heart also under physiological conditions^9^ due to intrinsic, spatially distinct ion channel expression and excitability of the myocytes (see *Figure 1*). Compared with the endocardium, subepicardial action potentials (APs) have a more prominent Phase 1 notch due to higher density of *I*_to_ as well as a shorter Phase 3 due to stronger *I*_Ks_ currents. In previous studies, some authors have proposed the existence of so-called mid-myocardial cells that appeared to take longer to repolarize due to weaker *I*_Ks_ but stronger late *I*_Na_ and Na ^+^ –Ca^2+^ exchange currents.^10–13^ These spatial gradients are complemented by temporal changes that have been associated with circadian patterns.^14^ Also, shorter temporal variations termed ‘periodic repolarization dynamics’ (PRD) have been proposed and associated with bursts of sympathetic activity inducing periodic low-frequency oscillations of repolarization that are independent of heart rate variability or respiratory activity. Elevated PRD has been associated with an increased arrhythmic risk.^15,16^ The anatomically heterogeneous innervation of sympathetic and vagal fibres and their autonomic inputs play a vital and complex role in the regulation of cardiac electrophysiology in general and repolarization in particular, including adaptation to physiological changes, e.g. in heart rate.^17–19^

### Pathophysiology

Under pathological conditions, changes in both spatial and temporal repolarization dynamics can be observed: abnormally *prolonged* repolarization duration, excessive *spatial dispersion* of repolarization duration arising from repolarization heterogeneity (globally or locally e.g. in areas of scar),^20,21^ and further accentuation by a non-uniform increase in beat-to-beat alternans of repolarization^22^ (see *Figure 1*). The latter may result in *discordant alternans* where local areas are out-of-phase with other adjacent regions in the beat-to-beat shortening and lengthening of AP duration.^23^ The magnitude and direction of spatial gradients in the transmembrane potential during repolarization phase may thus alternate and, if sufficiently steep, produce unidirectional conduction block and functional re-entry. Depending on the underlying substrate, this may result in ventricular fibrillation (VF)^22^ or monomorphic VT (in the presence of myocardial scar).^21^

The ionic basis of these abnormal repolarization dynamics at sites of myocardial scar and fibrosis has been reviewed^3,24–26^ although they are predominantly limited to post-infarct substrates: non-uniform down-regulation of potassium channels has been described to result in locally prolonged repolarization time and thus slow recovery of excitability^27^ and repolarization gradients towards adjacent areas. Abnormal conduction delay due to sodium current alterations causing a slower upstroke velocity in Phase 0^28^ and cellular uncoupling and/or abnormal gap junction connections^29,30^ also has been described to contribute to the dispersion of repolarization and refractoriness. Lastly, heterogeneous innervation may further (dynamically) aggravate conduction and repolarization gradients, as it has been best characterized for post-infarct arrhythmias^31^ and severe heart failure.^32^

### Functional vs. fixed conduction block in re-entrant ventricular tachycardia

**Figure 2:**
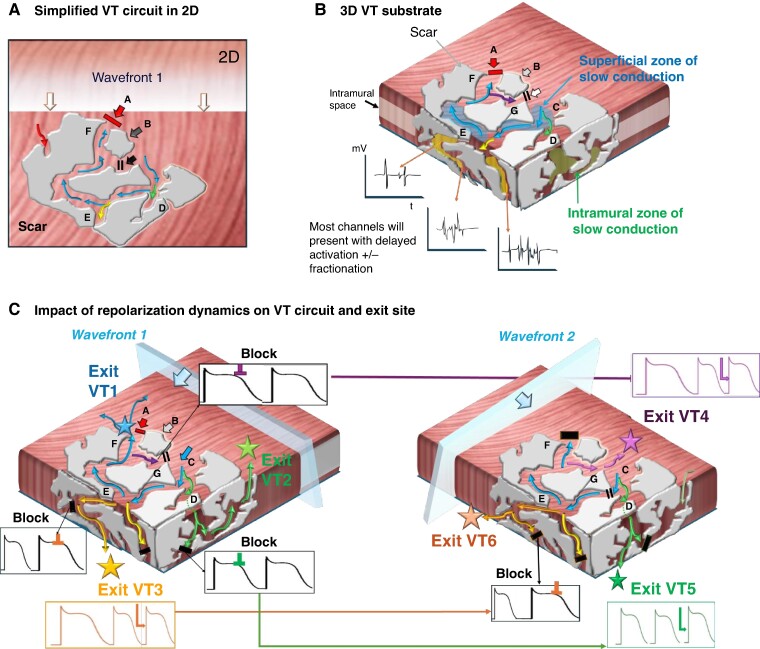
Modulation of VT circuits by repolarization dynamics: in presence of scar, both anatomical fixed and functional lines of block are likely to contribute to initiation and maintenance of VAs. The spatial and temporal gradients of repolarization may dictate and modulate the exact VT circuit. (*A*) A traditional simplified 2D schematic of a complex patch of scar (grey) with conducting channels. An approaching wavefront meets sites of functional block at ‘site A’ but ‘enters’ a conducting channel at ‘site C’. Sites D and E are bystanders. At ‘site G’, functional block occurs due to still refractory tissue at site C. In turn, by the time it arrives at ‘site F’, the opposing site A has regained excitability and the impulse conducts back and allows initiation of a re-entry (A = exit site). (*B*) Ventricular scar is a complex 3D construct with channels not only along the surface but also extending intramurally. (*B*) How the presumed ‘dead-end’ bystanders at sites D and E in panel (*A*) could lead towards intramural channels with equally delayed conduction that may result in alternative circuits either leading back to the original surface (green) or with exit sites to the opposing surface (yellow). Mapping may indicate late potentials and fractionation at all these channels. Bottom (*C*) Illustration of the role of repolarization dynamics in modulating VT exit site: different sites may display distinct APD durations and/or accentuated beat-to-beat variation: *Left*: Wavefront 1 may meet refractory tissue at some of the channels (black) yet timing may be right at others (e.g. blue exit VT1, green exit VT2, and yellow exit VT3). *Right*: A ‘different’ wavefront, e.g. change of direction or coupling interval and/or changes in autonomic tone, may unmask alternative VT circuits due to the dynamic changes in repolarization timing which could modulate the exit site. As schematically shown here, the wavefront now meets refractory tissue at the previous exits but allows the impulse to travel through to VT 4–6.

Originally, re-entrant VT was assumed to be predominantly the consequence of abnormal conduction slowing around a fixed barrier and conduction anisotropy, which may lead to a reduced safety factor for impulse transmission and risk of unidirectional block.^33–36^ The discussion about functional vs. fixed lines of block in scar-related VT in defining re-entrant circuits has been critically reviewed over the years,^37^ with some authors suggesting that source–sink mismatch^38^ and others that anatomic re-entry around *fixed* structural boundaries are the dominant causes of human re-entry VT that can be unmasked by differential pacing manoeuvres.^39^ Traditionally repolarization dynamics have not been assessed in these studies, yet in computational studies they were found to affect the VT re-entrant pathway and exit sites due to modulation of the site of conduction block.^40^  *Figure 2* shows an example of a complex scar with multiple channels and schematically illustrates how dynamic repolarization changes in addition to external factors (e.g. change activation wavefront direction and coupling interval as well as autonomic effects) may define the VT circuit.

It seems reasonable to assume that both fixed ‘static’ anatomical barriers and functional ‘dynamic’ block are present with intra- and interindividual differences in their relative contribution to the genesis of re-entrant VT. The fundamental differences in structural alterations across the numerous cardiopathies associated with VA including myocyte disarray, hypertrophy, interstitial fibrosis, and microvascular dysfunction in hypertrophic cardiomyopathy (HCM),^41^ fibro-fatty infiltration and replacement in arrhythmogenic cardiomyopathy (ACM),^42^ the variable extent of ischaemic scar and maturity of infarct with progressive lipomatous metaplasia,^43,44^ etc. are all associated with distinct electrophysiological remodelling patterns and characteristics. The dominant mechanism of VT may therefore vary and requires a full and integrated assessment of all contributing components.

## Practical aspects of repolarization mapping and re-entry vulnerability estimation

### How to generate high-density repolarization maps intra-procedurally: a step-by-step guide

**Figure 3:**
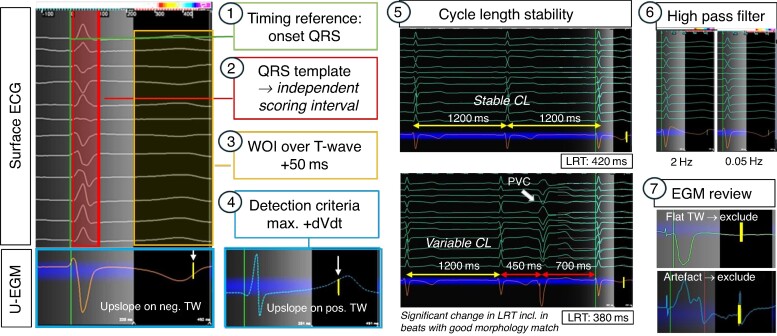
How to set-up repolarization maps. Setting up a repolarization map follows the same principles as for activation mapping, yet notable differences are in placing the WOI over the T wave, using the max. +dVdt as detection criteria and adjusting the high-pass filter to 005 Hz. Importantly, there are no automated feature to recognize distorted or flat TW, and therefore, careful manual review is required to exclude EGMs where no reliable LRT annotation is feasible (examples here: flat TW and artefact in TW). CL, cycle length.

High-density local repolarization timing (LRT) maps can be generated in any mapping system that offers an automated detection algorithm for maximal + dV/dt which can be employed for LRT annotation according to the Wyatt method.^45^ Currently available commercial mapping systems that offer maximal positive dV/dt as detection algorithm and therefore allow acquisition of repolarization maps are Ensite^TM^ X (‘max. +dV/dt’) and CARTO3^TM^ (‘upslope’). *Figure 3* illustrates the process of how to set up a repolarization map as follows:

1. Basic map set-up:

- Electrogram (EGM) type: The EGM type is set to unipolar.
- Morphology/score template: An independent scoring interval is used to define the surface QRS template beat (e.g. RV or LV paced or intrinsic QRS) of interest. The use of T wave templates is challenging and, in our experience, did not yield usable results.
- Cycle length stability: Thresholds for cycle length stability requires a strict margin (e.g. ±20 ms) due to the important cycle length dependency of repolarization dynamics.
- Timing reference: It is set to the beginning of the QRS, and thus, LRT annotation will correspond to the timings of the earliest myocardial activation to the local repolarization at the recording site and facilitates site-by-site comparison with local activation time (LAT) maps. Alternatively, a timing reference at the end of QRS can be used as per operator preference.

2. Specific set-up for repolarization maps:

- Window of interest (WOI): The WOI is placed from the end of the surface QRS to the end of the surface T wave + 50(−100)ms. The latter will allow to capture localized delayed repolarization which may not be reflected on a surface ECG T wave, yet a frequent limitation of current mapping systems (as they are designed for activation mapping) is that the length of time interval after the timing reference is restricted to 500 ms and therefore prevents annotation of locally very prolonged repolarization time (RT) sites.
- Detection criteria: This is set to detecting the maximal upslope (+dV/dt) within the WOI. This enables annotation of LRT according to the Wyatt method on the unipolar T wave of each EGM regardless of T wave polarity.
- Filter settings: The nominal unipolar high-pass filter is set at 2 Hz for commercial mapping systems. For repolarization mapping, it is vital to adjust this to 0.05 (−0.5 Hz) to prevent distortion of the T wave due to filtering
- Use of an inferior vena cava reference for unipolar EGM recording is advisable to optimize EGM quality.

3. Manual review of EGMs: Given the absence of dedicated repolarization mapping features that allow to automatically identify distorted T waves, unipolar EGMs require manual review to identify EGMs with noise, respectively, artefacts, e.g. R-on-T phenomena preventing accurate LRT annotation, that need to be (manually) excluded. Editing the repolarization map is of particular importance at sites of scar where frequent T wave distortions and/or flat T waves may challenge accurate annotation even with manual review.

**Figure 4:**
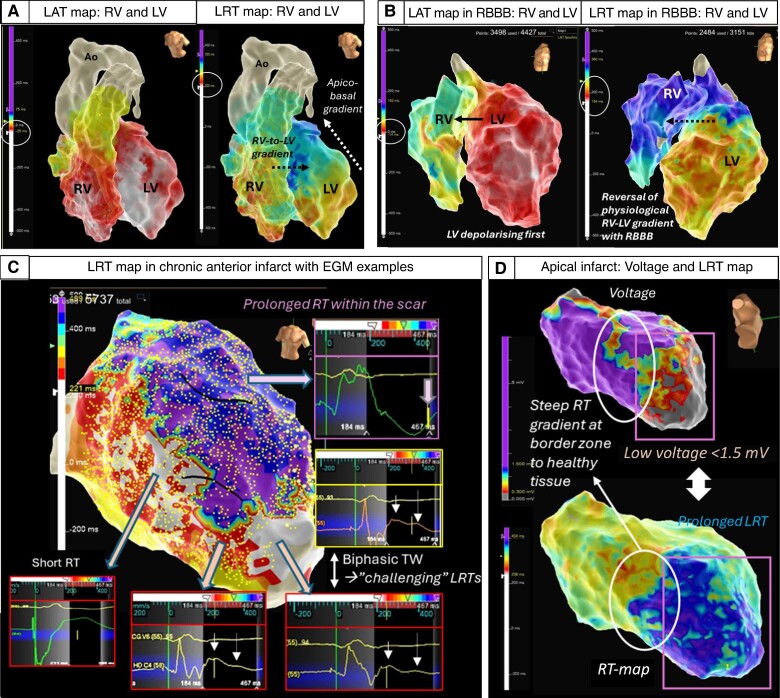
Examples of 3D repolarization maps in normal and diseased hearts. Top Left (*A*) A structurally normal heart assessed with high-density conduction and repolarization mapping demonstrating interventricular right-to-left and intra-ventricular apico-basal gradients (transmural epi-to-endocardial not shown).^11–13,46–48^ In the intact heart, the transmural repolarization gradient is thought to contribute comparatively little (13%) to the total dispersion of repolarization, whereas apico-basal, interventricular and anterior posterior gradients are thought to contribute the majority (87%)^49^ Top Right (*B*) DCM patient with mid-septal fibrosis and right bundle branch block showing an accentuation of the repolarization gradients and reversal of the right–left activation and repolarization sequence. Note: different colour scales for LAT and LRT. Bottom (*C*) High-density repolarization map in a patient with anterior myocardial infarction. Significantly delayed repolarization times in the anterior scar area (purple) with fractionated prolonged QRS in the unipolar EGMs reaching >220 s and pushing the repolarization time to the edge of the WOI that is possible to set up (maximum + 500 ms). Steep gradients can be appreciated at the border zone of the scar with biphasic T waves causing challenges for precise LRT annotation. (*D*) Low-voltage zone secondary to an apical infarct co-locates to areas of prolonged repolarization and steep gradients at the border zone.

*Figure 4* demonstrates examples of retrospectively generated repolarization maps with the presented approach in a patient with structurally normal heart (*Figure 4A*), a patient with dilated cardiomyopathy (DCM) and right bundle branch block (*Figure 4B*), and a patient with ischaemic cardiomyopathy with chronic anterior (*Figure 4C*) and apical infarct (*Figure 4*D).

#### Interpretation of local repolarization time maps

**Figure 5:**
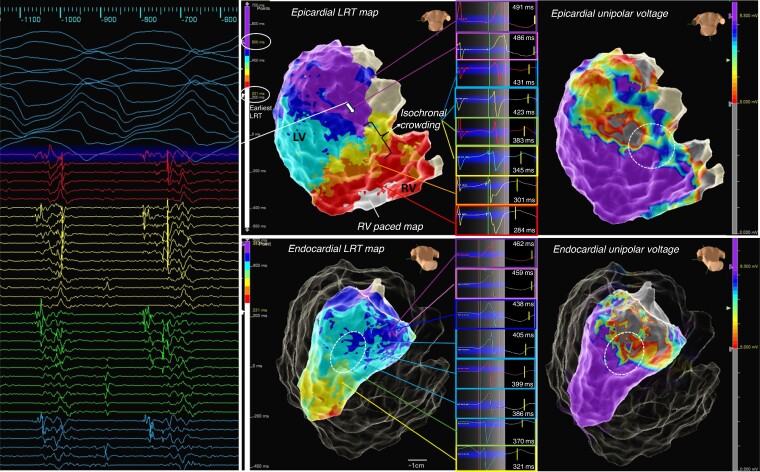
Example of iREM: a patient with left-sided ACM, inferolateral mid- and subepicardial scar in MRI, fast VT, and episodes of ventricular ectopy-triggered VF. High-density repolarization mapping was performed as described, with LRT map shown and total repolarization time displayed over eight isochrones (here 221 to 500 ms). A zone of isochronal crowding was identified at the inferior base at the border zone of the unipolar low-voltage area (white circle) allowing to quickly identify an area of abnormally steep repolarization gradients leading towards a zone of substantially prolonged repolarization corresponding to the area of scar as indicated in the voltage map. Bottom The corresponding LV endocardial LRT map equally displays a zone of isochronal crowding at the border zone in the mid-inferolateral segment albeit much more discrete. A fast VT (tachycardia cycle length 250 ms) was induced with loss of cardiac output, the HD grid overlaying the area of late repolarization adjacent to the iREM area recorded mid/end-diastolic potentials, with ATP the VT degenerated to VF and required DCCV. Activation sequence of the HD grid suggested conduction from area of late activation towards iREM.

**Figure 6:**
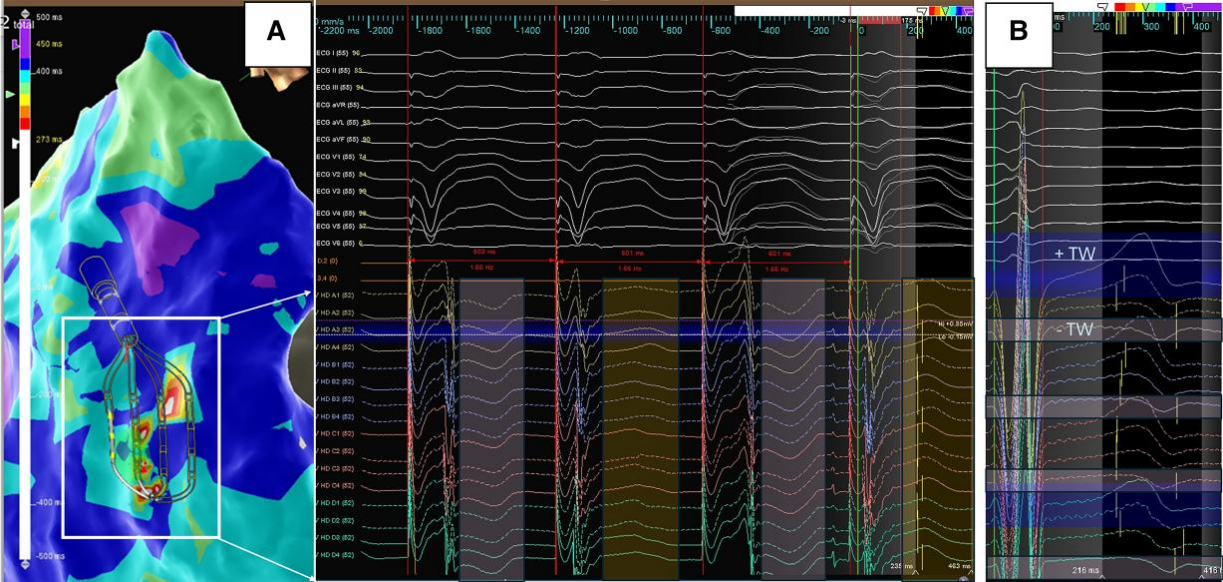
Example of T wave alternans displayed as ‘colour disarray’ on 3D map. Left (*A*) Discordant alternans of the T wave recorded by the HD grid. During stable cycle length and QRS morphology, alternating T wave polarity is recorded highlighting the challenges of sequential LRT mapping during ventricular substrate mapping. Right (*B*) T wave polarity change recorded across the splines of the HD grid (negative on Spline 1 to positive on Spline 4). Repolarization starts when the net of outward currents begins to exceed the inward current of the AP, which is a much slower process than the rapid activation and upstroke in Phase 0. A positive T wave reflects the current flowing towards the recording site, e.g. repolarization at this site occurs earlier, whereas the more negative, the later the repolarization occurs^51^—the high-density mapping catheter is able to record this changes and phenomenon during contact mapping.

High-density three-dimensional (3D) electro-anatomical mapping can provide an immediate ‘visual’ result that facilitates identification of local gradients and heterogeneities. A simple and intuitive approach for LRT interpretation is to adjust colour scales to cover the full repolarization time across the ventricle with user-defined number of isochrones [isochronal repolarization maps (iREM)] analogue to the previously proposed isochronal late activation mapping mapping.^50^ This will allow to identify sites of steep gradients which will manifest as isochronal crowding (see *Figure 5*) or ‘gaps’ (frequently due to changes in T wave polarity) as well as heterogeneity (visualized as ‘colour disarray’, see *Figure 6*). The latter may be distinguished from conduction mapping, where the nature of a propagating activation wavefront involves changes in the transmembrane potentials in neighbouring cells triggering a downstream AP to allow impulse propagation. In turn, repolarization may occur as localized events which may ‘only’ be modulated by neighbouring cells by electrotonic interaction. It has been suggested that in well-coupled cells with low intercellular resistance, a more prominent electrotonic interaction may decrease dispersion (‘homogenising effect’), whereas fibrosis may facilitate repolarization heterogeneity by reducing these electrotonic interactions.^26,52^

Equally, iREMs may differ from iLAMs in that their spatial and temporal stability may vary to a higher degree than deceleration zones. To further investigate this, multiple re-maps would need to be acquired which with current sequential mapping technology is not feasible during a clinical case.

### How to estimate re-entry vulnerability intra-procedurally: a step-by-step guide

**Figure 7:**
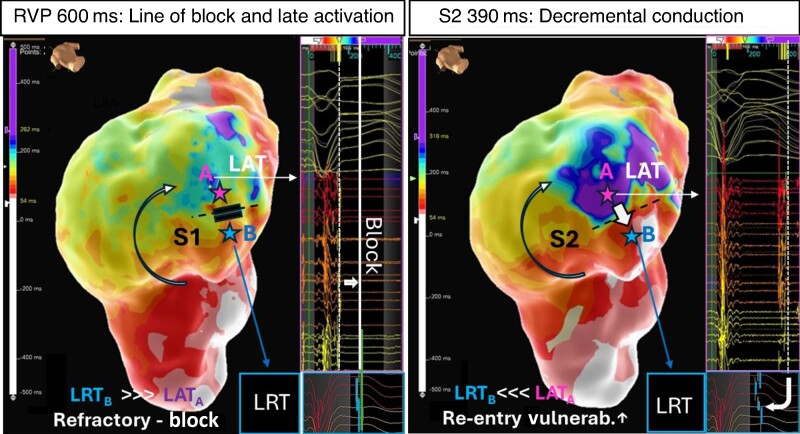
Concept of re-entry vulnerability index. Left A LAT map acquired during straight RV pacing at 600 ms (S1) shows a line of block and adjacent area of late activation on the anterior LV wall (blue/purple, point A). The adjacent area (point B) is still refractory at the time the late activating area is activated (LRT_B_ > LAT_A_). Right A critically timed sensed extra ‘S2’ with coupling interval at 390 ms elicits decremental conduction. The wavefront pivots around the line of block wavefront arriving now with a delay of over 300 ms in the late activating area and meets re-excitable tissue at the opposing side of the (unidirectional line of block (LRT_B_ < LAT_A_) and thus predisposes to re-entrant tachycardia.

Complementary to the assessment of repolarization gradients and heterogeneity, the LRT map may then be combined with their corresponding conduction maps to identify re-entry vulnerable sites associated with putative VT circuit sites during baseline rhythms. Integrating repolarization timing to existing substrate mapping approaches may allow for more specific ablation strategies: if deceleration zones of conduction delay demarcate the proximal limb forming the line of block to enable re-entry to be initiated, the key question would be how close these zones are to sites of short repolarization timing where the wavefront encounters re-excitable tissue. It would be this shortest RT site which would be expected to be critical to target with ablation and could minimize the extent of the required ablation lesion set. To investigate this question, previous experimental studies attempted to quantitatively assess likelihood of excitation wavefront–waveback interactions between two points across a line of conduction block following a premature stimulus, providing valuable information to potentially guide ablation when haemodynamically stable VT cannot be induced—this metric was termed the ‘RVI’^8,53^ and the concept illustrated in *Figure 7*. Using contact mapping data, sites of late activation (AT_L_) adjacent to sites with relative early, i.e. short repolarization time (RT_E_, to allow for re-excitability), can be used to calculate RVI on a point-by-point basis. Each point is allocated a numeric RVI defined as the minimum difference between RT_E_ at the proximal site and AT_L_ at the distal site within a given radius. This approach showed promising results in retrospective studies estimating RVI offline,^54,55^ but translating it into a clinically useful functional mapping strategy has so far been limited by a lack of reliable tools to compute RVIs intra-procedurally.

To overcome this barrier, we recently proposed the use of a ‘visual re-entry vulnerability index’ (RVI_V_) that allows to combine existing activation metrics (i.e. late activation areas, deceleration zones, lines of block) and visually identify re-entry vulnerable zones corresponding to putative VT sites without requirement of additional mapping tools or software features.^56^ Visual re-entry vulnerability estimation can be performed in three steps:

1. Local activation time maps using last deflection detection are generated to identify zones of deceleration and late activation.
2. High-density local repolarization time maps are generated as outlined above using the existing mapping segments recorded for the LAT maps (employing ‘Turbo Map’ or ‘Re-Map’ features of the mapping system) in that both LAT and LRT measurements are based on the same EGM data set. Turbo maps run with 10 times the speed of the original acquisition and thus are time-efficient enough to be employed intra-procedurally.
3. Local activation time maps and LRT maps are displayed side-by-side. Zones of early (e.g. in example below ‘white/red areas’) repolarization, i.e. shortest RT, overlapping or directly adjacent to tagged zones of late (dark blue/purple) activation are considered re-entry vulnerable (‘RVI_V_ zone’). Degree of vulnerability may further be quantified by assessing the temporal (timing difference) as well as spatial relationship (distance in mm) of early repolarization time zones (RT_E_) to late activation zones (AT_L_) and a numeric RVI calculated (=RT_E_ − AT_L_) to support the visual assessment. In previously reported studies, the maximum distance between RT_E_ and AT_L_ for RVI calculation was 20 mm.^55^ We suggest that within that radius, point pairs with a maximum time differences between AT_L_ and RT_E_ EGMs of <100 ms could be considered re-entry vulnerable accounting for possible conduction slowing of as low as 0.2 m/s in diseased tissue as reported previously in preclinical studies.^57^

#### Interpretation of re-entry vulnerability index maps

**Figure 8:**
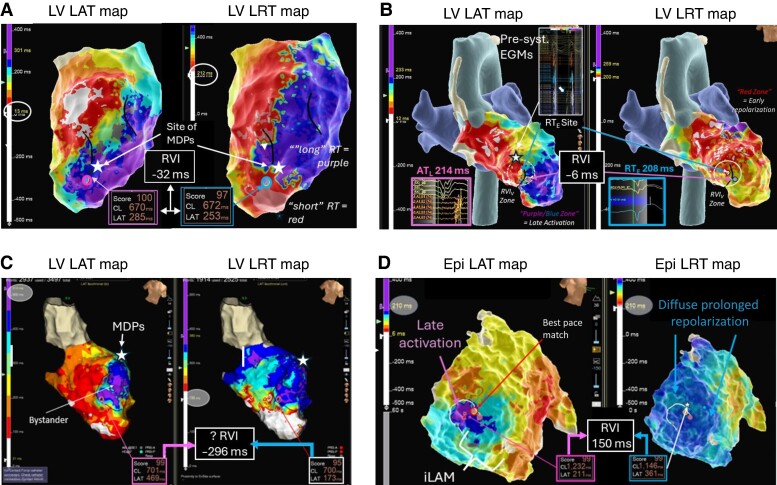
Example of RVI_V_ mapping in ischaemic and non-ischaemic substrates. (*A*) Ischaemic LV substrate. Evidence of extensive areas of late activation with a broad deceleration zone (marked with black lines) in LAT map. Corresponding LRT map suggests a very steep gradient at the border zone of the scar large anterior scar and a visual RVI zone (purple and blue circles) is identified towards the anterior apical segment with calculated RVI −32 ms within AT_L_ and RT_E_ < 5 mm (‘very high vulnerability’). The white star indicates the location of mid-diastolic potentials during the VT. (*B*) Burned-out HCM: Again extensive area of scar in the LV apex with late activation and a deceleration zone rotating around a line of block in the septo-apical segment. An adjacent zone of early repolarization allows to locate a visual RVI zone (white dashed circle) with estimated RVI of −6 ms. During VT mapping, presystolic EGMs were recorded just slightly superior to the RVI. (*C*) False-positive RVI in an ischaemic substrate with anterior LV aneurysm with excessive delay of >400 ms into the aneurysm sack. Highest re-entry vulnerable zone was identified at the mid-anteroseptal edge of the aneurysm with RVI −296 ms across a line of block. Re-entry around the neck of the anterior-basal edge of the aneurysm was mapped and terminated with ablation (not shown here). Ablation at the base of the aneurysm neck electrically isolated the entire aneurysm confirming a true anatomical (as opposed to functional) line of block and unmasking the original RVI1 site as false positive. (*D*) ‘Unsuccessful’ RVI mapping in a patient with arrhythmogenic right ventricular cardiomyopathy (ARVC) and VT. Zone of deceleration and late activation on RV free wall of epicardial LAT map but no adjacent sites of early repolarization on LRT map. The possibility of an intramural re-entry vulnerable site needs to be considered, and possible combining endo- and epicardial LAT and LRT maps may increase the success rate of RVI mapping in these patients.

The lower, respectively negative, the estimated RVI, the higher the vulnerability for re-entry. *Figure 8* shows four examples of RVI mapping in ischaemic and non-ischaemic cardiomyopathies with reference to activation mapping and/or pace mapping exit sites. In a previously reported proof-of-principle retrospective case series of 20 patients with scar-related VT who had undergone VT ablation, this approach identified in 17/20 (85%) patients at least one re-entry vulnerable zone with manually calculated RVIs estimated to a median of 32.0 ± 100 ms and shortest distance of any RVI_V_ on map to functionally critical sites during VT was 8.2 ± 7.1 mm (range 0–33 mm).^56^ Interestingly, in that case series in two cases, the RVI_V_ closest to the clinical VT exit was not the RVI_V_ with the highest ‘numeric’ re-entry vulnerability.

## The challenges and limitations of repolarization mapping

Despite the promising new insights high-density repolarization mapping may offer to clinicians, several practical issues as well as intrinsic features of repolarization need to be addressed, respectively considered, to further facilitate widespread use of clinical application of repolarization mapping:

### Spatial and temporal dynamicity of human repolarization

The dynamic character of the repolarization phenomena poses a fundamental challenge for sequential LRT assessments. The pronounced cycle length dependency^58,59^ makes time-consuming beat-by-beat LRT measurements susceptible to error if RT pattern (e.g. gradients/heterogeneities) is being assessed (see *Figures 5* and *6*). Numerous internal or external factors (e.g. episodes of VT, ATP, or cardioversion resulting in possible myocardial ischaemia and stunning; catheter-induced iatrogenic extra beats which frequently occur during ventricular contact mapping) may have a profound effect of repolarization dynamics and memory effects and/or may distort T waves (e.g. R-on-T). Autonomic influences on repolarization dynamics, choice of anaesthetic and/or even variable depth of anaesthesia throughout the procedure, may also affect LRT during acquisition undermining the value of a sequentially recorded LRT maps.

Even in stable cycle length, the intrinsic beat-to-beat changes of AP duration, while being a marker of arrhythmogenicity and thus diagnostically important, are difficult to record and annotate with current mapping technology.

### Spatial inaccuracy and noise susceptibility of unipolar EGMs

The wide ‘field of view’ of unipolar EGMs poses another challenge if high spatial resolution and accuracy are needed. The question remains how closely these ‘local RTs’ spatially relate to the often complex, low-amplitude fractionated, and/or late potentials visible using omnipolar EGMs acquired with dedicated multipolar mapping catheters with small electrodes and tight interelectrode spacing. The fundamental differences in these EGM types hamper a truly accurate combination of local AT and RTs precisely at the sites of greatest interest. In our pilot data set, we found that indeed ‘short’ unipolar RT and ‘late’ omnipolar AT may overlap on the same surface. Yet, the use of omnipolar EGMs is superior to unipolar EGMs in identifying deceleration zones and lines of block and there is so far no alternative to unipolar EGMs for repolarization mapping.

This is further complicated by a significant proportion of distorted and/or flat T wave in unipolar EGMs, particularly at sites of scar, or R-on-T phenomena due to spontaneous or iatrogenic extra beats or artefact. This can prevent any reliable RT annotation to begin with and require a high number of EGMs to be excluded from further analysis. The susceptibility of unipolar signals to noise, even with the use of dedicated inferior vena cava electrode as reference, is another limitation. While manual re-annotation is possible in some instances, the manual identification of the steepest point on the upslope of the T wave may easily introduce errors in the range of 20–50 ms. In our previously reported pilot data set^56^ of 20 patients with scar-related VT, around 20–25% of intracardiac EGMs were unsuitable for LRT annotation.

Novel promising techniques using orthogonal electrode configurations have already been proposed^60^ and may overcome some of the challenges highlighted above. If shown to be accurate and reliable, this may be a revolutionary step forward for intracardiac mapping and ventricular substrate characterization to a similar degree as the introduction of dynamic pacing protocols to elicit decremental conduction and identification of deceleration zones to identify ablation targets.

### Lack of clinical repolarization mapping tools

Contemporary EP mapping systems are ill-equipped for repolarization mapping due to the exclusive focus on conduction metrics. Our proposed workflow for repolarization mapping involves a number of adapted ‘custom-made’ steps for this purpose and importantly in some cases substantial manual review. Some of these challenges may be easily addressed in available mapping systems (e.g. filtering of distorted T waves, extension of theWOI, automation of RVI estimation). Others may require new approaches including hardware with mapping technologies offering global, simultaneous EGM acquisition as well as novel LRT definitions, e.g. based on omnipolar technology.

A combined endo-epicardial assessment of conduction and repolarization metrics may improve re-entry vulnerable site identification to better reflect the 3D nature of the arrhythmogenic substrate. Yet, true mid-myocardial substrate hidden intramurally may still be missed and new mapping technologies are required to address this short coming.

## Conclusions

High-density repolarization mapping is feasible and may provide novel insights in the individual arrhythmogenic substrate and support identification of re-entry vulnerable zone without the need for VT induction. It represents the natural next step in the evolution of ventricular mapping strategies following the important progress and efforts in recent years of defining more specific, physiology-based ablation targets to improve patient outcomes. Yet, important limitations and challenges exist, and as of today, it remains investigational with no prospective data supporting its routine use in clinical ablation procedures. Novel mapping technology that could afford simultaneous global assessment of repolarization dynamics and dedicated automated repolarization mapping features in electro-anatomical mapping systems are required to further facilitate integration of this important metric in contemporary mapping workflows and evaluate in prospective studies. Equally, the definition of local repolarization times on unipolar EGMs poses challenges due to the lower spatial inaccuracy and susceptibility to noise and artefacts. Innovative approaches for defining LRT derived from orthogonal bipoles and multielectrode arrays may address some of the important shortcomings of current RT mapping and await validation.

## Data Availability

Data are available on request due to privacy/ethical restrictions.
